# Morphology of SiO_2_ films as a key factor in alignment of liquid crystals with negative dielectric anisotropy

**DOI:** 10.3762/bjnano.7.167

**Published:** 2016-11-17

**Authors:** Volodymyr Tkachenko, Antigone Marino, Eva Otón, Noureddine Bennis, Josè Manuel Otón

**Affiliations:** 1 CNR-ISASI and Physics Department, University of Naples Federico II, Via Cinthia Monte S. Angelo, 80126, Naples, Italy; 2 CEMDATIC, E.T.S.I. Telecomunicación, Universidad Politécnica de Madrid, Avda. Complutense 30, 28040 Madrid, Spain,; 3 Institute of Applied Physics, Military University of Technology, 00-908 Warsaw, Poland

**Keywords:** anisotropy, ellipsometry, liquid crystal alignment, morphology, thin film

## Abstract

Control of liquid crystal (LC) orientation using a proper SiO_2_ alignment layer is essential for the optimization of vertically aligned nematic (VAN) displays. With this aim, we studied the optical anisotropy of thin SiO_2_ films by generalized ellipsometry as a function of deposition angle. The columnar SiO_2_ structure orientation measured by a noninvasive ellipsometry technique is reported for the first time, and its morphology influence on the LC alignment is demonstrated for large deposition angles.

## Introduction

Thin films of SiO_2_ and SiO*_x_* obtained by oblique deposition are commonly used as alignment layers for liquid crystal (LC) displays [1–7]. Several alignments can be induced in nematic LCs, including the homeotropic employed in VAN displays. However, the LC director of actual VAN displays is never exactly vertical. Even with no applied voltage, there is always a small angle between director and the normal to the alignment surface, called the pretilt. The control of pretilt in VAN cells is essential in many applications, as it affects display contrast and response time [2,6–7].

Pretilt depends on a number of factors such as chemical interaction forces between LC molecules and an alignment film, dielectric anisotropy of the LC, and film morphology. Many authors have reported that LC alignment can be controlled by choosing the deposition angle, α, defined as the angle between the SiO_2_ vapor beam and normal to the substrate [2–6]. At α < 40° LC molecules with positive and negative dielectric anisotropy, Δε, tend to align homogeneously and homeotropically, respectively. Such behavior can be explained by induced dipole–dipole interaction between LC and a smoothed oxide surface derived from van der Waals forces [3,8]. When α increases from 60° to 85°, the preferred orientation of LC with positive Δε switches from orthogonal to parallel to the plane of particle incidence. Moreover, two-fold alignment domains can appear with two easy axes symmetrical with respect to that plane [4,8]. Amosova et al. [5] explained switching in LC alignment in terms of the length of the crystallites forming relief of the aligning surface and surface wetting by particular LC. In the case of negative Δε, LC alignment only takes place in plane of particle incidence [3–4], defined mainly by film morphology.

The most amazing morphological feature of obliquely deposited polycrystalline films is its columnar structure, which appears to be due to a self-shadowing effect [9] and results in columns tilted with respect to the substrate normal. The empirical "tangent rule" has been proposed [10] to describe the connection between the deposition angle and the column tilt. A number of theoretical models has been proposed to understand formation of the columnar structure, such as a ballistic shadowing model giving the so-called "cosine rule" [11], a continuum model taking surface diffusion into account [12], and a model that accounts for the tendency of the columns to fan out during deposition [13]. The analytical formulas obtained within these models provide a first estimation, but fail when applied to wide ranges of deposition angle and film materials. Some numerical approaches can improve agreement with experiment taking into consideration surface diffusion and the angular broadening of the deposition flux [14–15].

Obliquely deposited SiO_2_ films are of special interest in photonics because of porosity dependence on the deposition angle, and consequently low and controllable refractive index. Many research groups have studied such films with ellipsometry techniques [16–18]. Some of them [16–17] investigated porosity and refractive index without taking into account birefringence while Gospodyn et al. [18] studied anisotropic refractive index as well. However, information on the index ellipsoid orientation of obliquely evaporated SiO_2_ films is still missing.

The connection between SiO_2_ column tilt and pretilt of LCs is not clarified yet. Most papers focused on LC alignment have not properly studied the morphology of the SiO_2_ films, and particularly, column tilt in a wide range of deposition angles [2–5]. Numerous experimental results reported in the papers, which are focused solely on SiO_2_ films, cannot help because of strong dependence of film properties on deposition conditions (e.g., deposition technique, gas composition and pressure in a chamber, deposition rate, substrate temperature, impurities) [19].

In this work, we investigate the morphology of obliquely deposited SiO_2_ films and its effect on alignment of LCs with negative Δε. We kept the same deposition conditions while preparing SiO_2_ samples and SiO_2_ alignment layers for LC cells. The optical anisotropy of all samples was characterized by the ellipsometric technique. The SiO_2_ column tilt and LC pretilt are obtained from the orientation of corresponding index ellipsoids. We report all measured parameters as a function of the deposition angle. Some of the results are compared with the experimental data and simulations from literature.

## Experimental

### SiO_2_ films and LC cells

SiO_2_ thin films were deposited by electron-beam evaporation on indium tin oxide (ITO) coated glass substrates (Glasstone) using a Vacudel 300 Telstar. The pressure in the chamber and substrate temperature were 5.3·10^−4^ Pa and 330 K, respectively. Granules of pure SiO_2_ (99.99%) were used as a source of evaporated material. The distance between the source and substrate was 75 cm to minimize angular spread of deposition on the substrate to about 1.5°. The samples were deposited at deposition angles of 0°, 30°, 40°, 50°, 60°, 70°, 75°, 80°, 82°, 84°, and 86° (i.e., grazing incidence). We used a deposition rate of 1 Å/s, as measured at normal incidence, to ensure a constant columnar structure through the film. The nominal thickness of the SiO_2_ films was increased from 60 nm for α = 0° to 400 nm for α = 86° because the structure becomes less packed at increasing deposition angles [2]. Thick and homogeneous layers are required in ellipsometry studies of low-index films.

For LC pretilt measurements, the SiO_2_ layers were kept to approximately 10 nm to minimize their optical influence on the proper characterization of LC layer. The VAN cells were comprised of two SiO_2_-coated substrates of opposite deposition directions, 4 μm spacers, and a Merck MLC6608 nematic LC with Δε = −4.2.

### Ellipsometry

A variable angle spectroscopic ellipsometer (VASE) from J.A. Woollam Co., Inc. was used for optical characterization. The six generalized ellipsometry (GE) parameters Ψ, Ψ_sp_, Ψ_ps_, Δ, Δ_sp_, and Δ_ps_ are linked to the Jones matrices of the reflected (**J^r^**) or transmitted (**J^t^**) beam via following equations [20]:

[1]
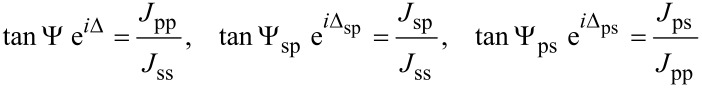


where *J*_pp_, *J*_ss_, *J*_sp_, and *J*_ps_ are the corresponding elements of the matrices


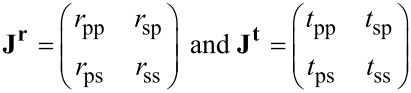


with *r*_pp_*, r*_ss_*, r*_ps_*, r*_sp_ (*t*_pp_*, t*_ss_*, t*_ps_*, t*_sp_) representing the reflection (transmission) coefficients for p*-*, s-, and cross-polarizations, respectively.

GE is a technique that measures thickness and dielectric constants of anisotropic thin films, being strongly sensitive to orientation of the index ellipsoid. The quantitative information for the desired physical parameters can be obtained by using inversion of the experimental data, as ellipsometry does not directly measure film parameters, but the integral optical response of the whole stack. To extract the film parameters, it is necessary to draw up a multilayer optical model in which the unknown parameters – such as thickness or optical constants values – are adjusted until the model best fits the experimental data. The number of existing studies on ellipsometry and LC confirms this technique as a reference for such materials. The measure of the optical constants [21], tilt distribution and anchoring strength [22] in nematic LC cells is well reported. The integration of VASE with other techniques [23] has also been investigated to improve the reliability and accuracy of the optical constants measurement, in an effort to reduce the critical dependence of the multiparameter fitting procedure from the initial guess.

**Figure 1:**
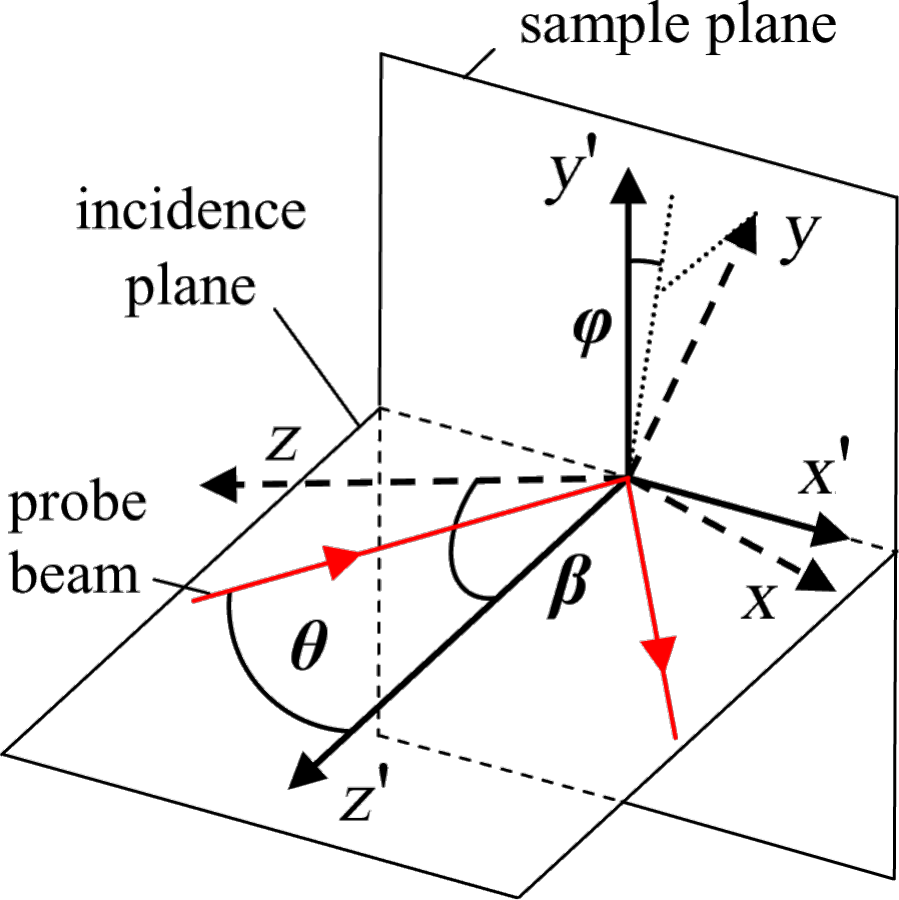
Incidence of the probe beam on the sample and the laboratory (*x*',*y*',*z*') and the sample (*x*,*y*,*z*) coordinate systems.

Figure 1 schematically shows the probe light beam incident on the sample at angle θ. Two coordinate systems are used in this optical model. The first one is the laboratory system, whose coordinates (*x*',*y*',*z*') are chosen with the *y*' axis normal to the incidence plane, and the *z*' axis normal to the sample plane. The second one is the sample reference system, whose coordinates (*x*,*y*,*z*) diagonalize the anisotropic permittivity tensor. These two systems are related to each other by two Euler angle rotations: by angle φ about the axis *z*' and by angle β about the axis *x*. β is a tilt of the axis *z* from the normal to the sample plane, while φ is the angle between the *y*' axis and the projection of the axis *y* on the sample plane. The samples were oriented such that the SiO_2_ deposition angle was in the *y*'–*z*' plane (φ ≈ 0°).

In order to decrease the number of fitting parameters, the glass substrate and the ITO coating were characterized first. The optical properties of these materials were kept constant during the fit procedure for SiO_2_ films and LC cells, as usual for ellipsometric study of multilayers [6,21–23]. To characterize the SiO_2_ films, a 3-step strategy was applied as adopted elsewhere [6]:

1. Reflection GE parameters are measured at 50°, 60°, and 70° in the wavelength range λ = 400–1700 nm. These data are mostly sensitive to the optical constants and thickness of the anisotropic layer.
2. Transmission GE parameters are measured at λ = 1200 nm varying θ from −15° to 60°. The off-diagonal elements of the Ψ and Δ matrices are strongly affected by the orientation of index ellipsoid (angles β and φ) but also by birefringence and film thickness.
3. The optical model is adjusted until the best fit to the experimental data is obtained, revealing anisotropic refractive index, film thickness and orientation of index ellipsoid.

During LC cell characterization, reflection GE parameters were measured at θ = 56.5°, close to the Brewster angle for glass–air interface at the working wavelength range; in this way, reflections from glass substrates are reduced. These reflections could decrease the degree of polarization of the beam, and consequently, the accuracy of the measurements. The transmission GE parameters were obtained in the same way as for SiO_2_ films.

The dielectric function ε_j_ of porous SiO_2_ layers was described using the effective media theory of Bruggeman [24], generalized for ellipsoidal inclusions of two components which are equally oriented and randomly dispersed [25]:

[2]
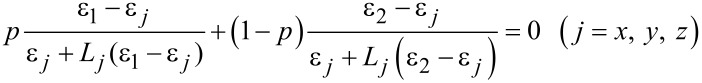


Here *p* is the porosity (volume fraction of pore), *ε*_1_ = 1 is the permittivity of air, and *ε*_2_ is the permittivity of SiO_2_ [26]. *L_j_* are the adjustable depolarization factors for the three main axes of the ellipsoidal inclusions, describing the effect of inclusion shape on the anisotropic dielectric function. The depolarization factor dependence on light polarization is easy to demonstrate for the case of spheroidal inclusions: *L_x,y,z_* = 1/3 for spheres; while 0 < *L*_z_ < 1/3 for light polarized parallel to the major axis *z* of a prolate spheroid and 1/3 < *L_x,y_* < 1/2 for orthogonal polarizations. The main semi-axes of the index ellipsoid are defined by *n_x_* = √ε*_x_*, *n_y_* = √ε*_y_* and *n_z_* = √ε*_z_*. The orientation of the index ellipsoid is determined by the angles β = β_SiO2_ (SiO_2_ column tilt) and φ = φ_SiO2_.

The LC layer was considered as a uniaxial optical medium, whose optical axis was defined by the angles β = β_LC_ (LC pretilt) and φ = φ_LC_. The ordinary and extraordinary refractive indices were described by the Cauchy dispersion formula.

Porosity, *L_j_*, β_SiO2_, β_LC_, thicknesses of SiO_2_ and LC layers and the Cauchy parameters were found by experimental fits. Even though the values of φ_SiO2_ and φ_LC_ were less than 1°, we fit them to improve accuracy of the desired physical quantities.

## Results and Discussion

**Figure 2:**
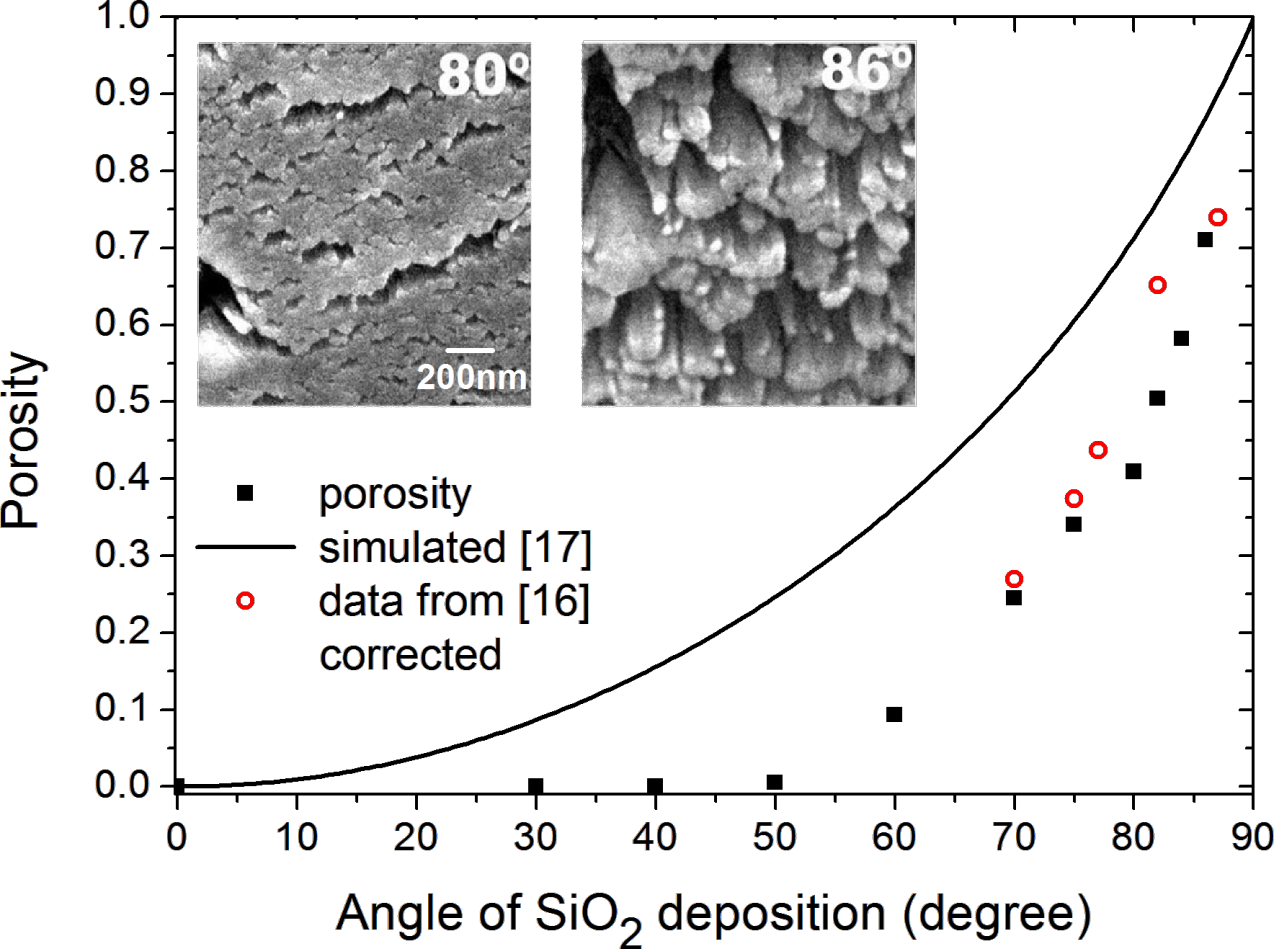
Porosity of SiO_2_ films vs angle of deposition (filled squares). Also shown are corrected data from [16] (empty circles) and the curve simulated in [17]. The inset shows FESEM top-view images of SiO_2_ samples for α = 80° and α = 86°.

The porosity of SiO_2_ films is close to zero for α less than 50° and increases with α up to 0.71 at α = 86° as shown in Figure 2. The inset shows FESEM top-view images of SiO_2_ samples for α = 80° and α = 86° [2]. As one can see, the SiO_2_ morphology becomes less packed and a columnar structure appears to be tilted from the viewing direction when the angle of deposition increases.

Yang et al. [16] calculated the film porosity from the measured isotropic refractive index *n* using the formula *p* = 1 – [(*n −* 1)/(ε_2_ − 1)] which overestimates porosity values by a factor 3*n*^2^/(2*n*^2^ + 1) as compared with Equation 2. In Figure 2 we present their data corrected to be consistent with Equation 2. Moreover, we present the theoretical curve [17] obtained on the basis of the ballistic model taking into account superficial diffusion of SiO_2_ particles during the deposition process. According to that model, the influence of superficial diffusion diminishes with α growth so that the porosity derivative increases with α approaching 90°. That explains the dramatic change in the observed film morphology for α = 80° and α = 86°.

Both experimental data are much below the theoretical curve which can be caused by the presence of water within hydrophilic SiO_2_ pores which is difficult to control in ex situ film characterization [18–19]. The presence of water results in an overall increase of the refractive indices and decreases porosity as compared with theoretical predictions. Note that porosity values are necessary for making the adequate ellipsometric model of SiO_2_ film but they do not affect β_SiO2_ data which are our main objective.

The case of zero porosity is also in contrast to the theoretical curve for deposition angles less than 50°. This is due to SiO_2_ islands coalescing without forming distinguished columns when surface diffusion overcomes the shadowing effect [19]. The film structure appears to be "pebble dash-like" [2] with a density approaching that of the bulk material.

**Figure 3:**
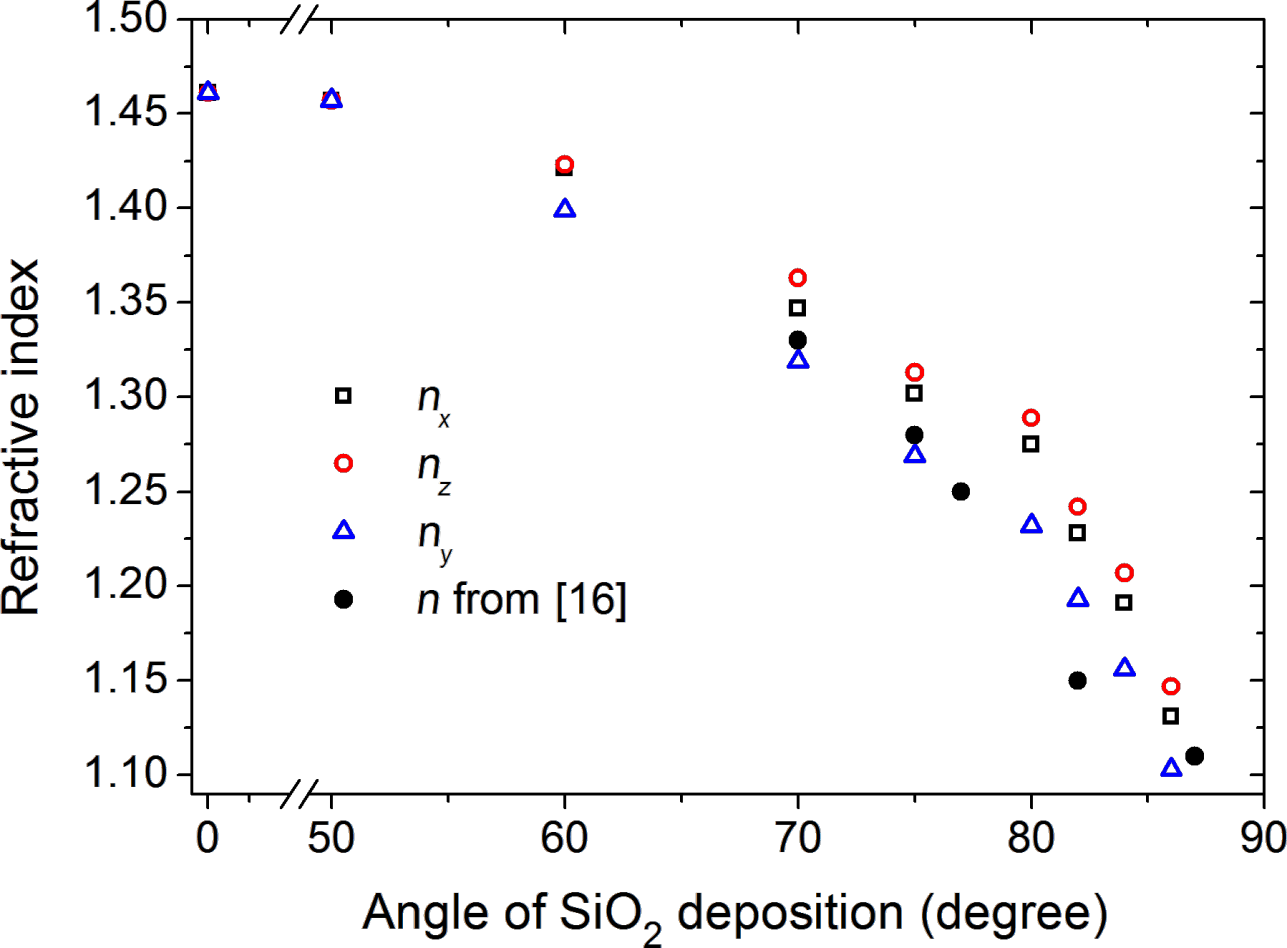
The anisotropic refractive index (nonfilled symbols) of porous SiO_2_ at λ = 532 nm vs angle of deposition. Also shown is the isotropic refractive index from [16] (filled circles).

Figure 3 shows the three components of the SiO_2_ anisotropic refractive index at λ = 532 nm as a function of the deposition angle.

At α < 50°, the depolarization factors are equal, resulting in the isotropic film with the refractive index of pure SiO_2_. For higher α, the depolarization factors correspond to ellipsoids with major axis along the *z* axis and the minor axis along the *y* axis. For example, at α = 86°, *L_x_* = 0.250, *L_y_* = 0.685, and *L_z_* = 0.065 with the error of fit ±0.003. Therefore, the film structure is constituted by SiO_2_ columns, which are directed along the *z* axis and have cross-section elongated in the *x* direction, in agreement with the corresponding FESEM images. The refractive indices decrease, as expected, with α, as the film density decreases, showing always the same relation *n_z_* > *n_x_* > *n_y_*. Birefringence increases with α and reaches values *n_z_* − *n_y_* = 0.042 and *n_z_* − *n_x_* = 0.014 at α = 86°. The fitting error is ±0.003 and ±0.01 at high and low α, respectively. The refractive index, *n*, obtained in the isotropic approach [16] is shown also to be somewhat less than our averaged data (*n_x_*+*n_y_*+*n_z_*)/3 (not shown). Note that Gospodyn et al. [18] reported the anisotropic refractive index to be about 5% less than our data, probably due to less water in the pores.

**Figure 4:**
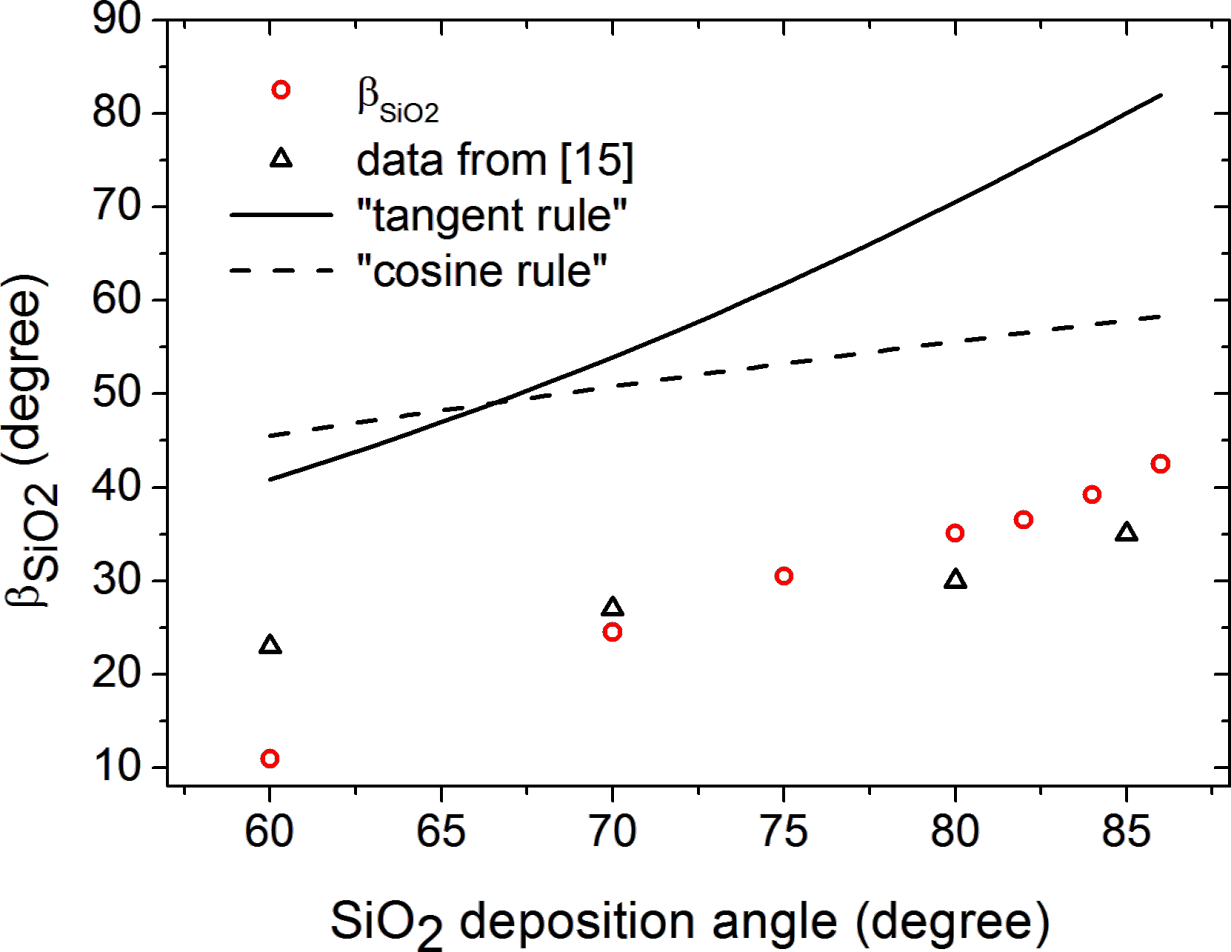
SiO_2_ column tilt vs the SiO_2_ deposition angle. The experimental data from [15] and the curves simulated according to the "tangent rule" [10] and "cosine rule" [11] are shown for comparison.

The SiO_2_ column tilt is shown in Figure 4 as a function of the deposition angle. We were not able to measure β_SiO2_ for deposition angles less than 60° because of very low film anisotropy. Greater angles of deposition lead to larger column tilt. The theoretical curves obtained by the "tangent rule" (tan β = 0.5 tan α), the "cosine rule" (β = α − sin^−1^((1 − cos α)/2)), and experimental data from [15] are shown for comparison. The theoretical curves do not agree with experimental data as they do not account for surface diffusion.

To the best of our knowledge, the columnar SiO_2_ structure orientation is measured for the first time by a noninvasive ellipsometry technique, which has evident advantages with respect to SEM imaging which implies film cleaving in plane of particle incidence [15–16].

**Figure 5:**
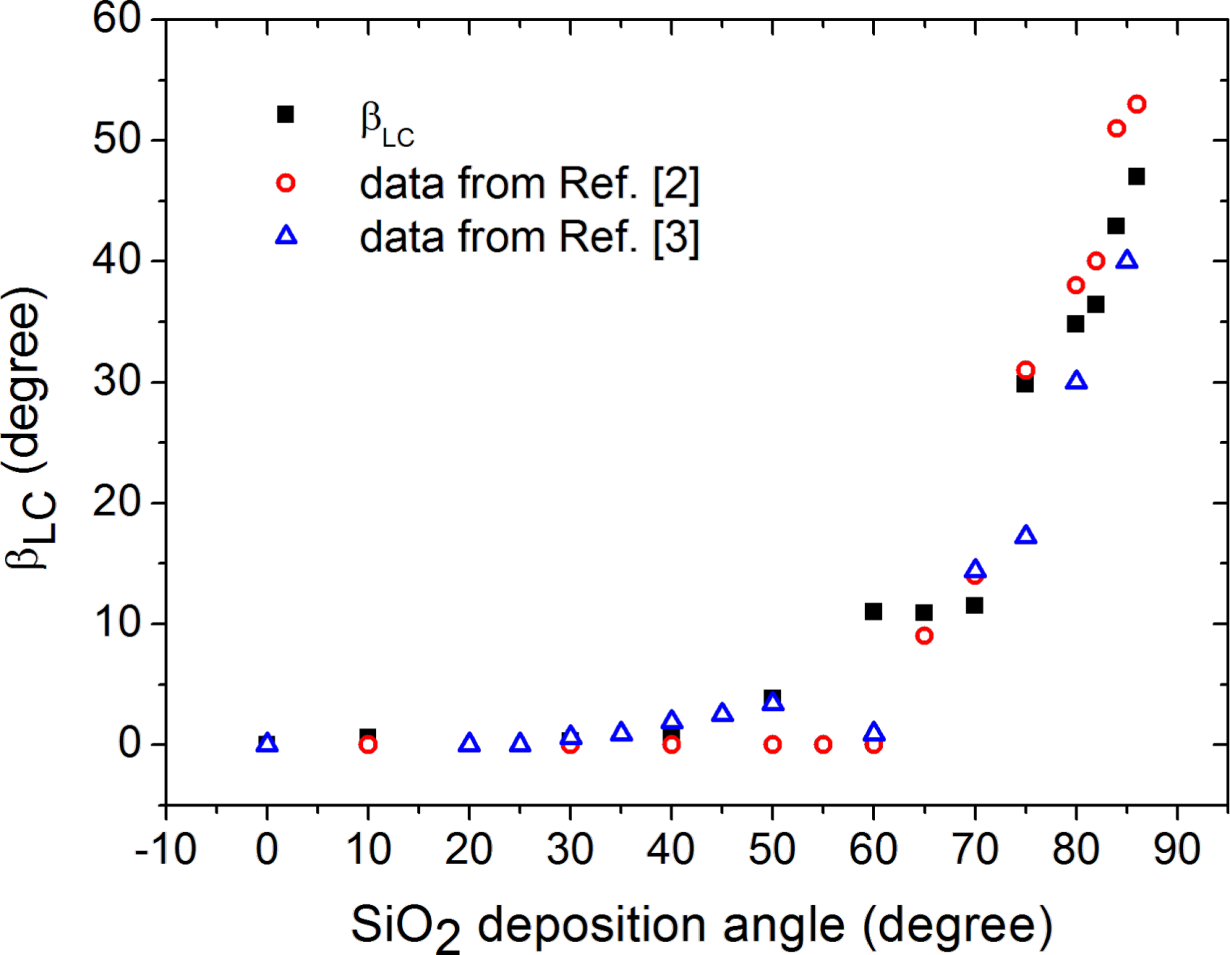
LC pretilt vs the SiO_2_ deposition angle. The experimental data from [2] and [3] are shown for comparison.

The LC pretilt is plotted in Figure 5. The fitting error of the β_LC_ data is about 1°. We show for comparison the experimental data from [2], where the same LC was used, and data from [3] for an MLC95-465 (Merck) liquid crystal with Δε = −3.8.

According to our data and that reported in [3], pretilt is close to zero at α < 30° and exhibit growth with α at 30° < α < 50°, while the data reported in [2] reveal growth starting from α = 60°.

In the range 50° < α < 75° high discrepancy is observed between the data of different authors, as a transition occurs from "pebble dash-like" structures to columnar structure of SiO_2_ film [2]. We believe that LC anchoring becomes spatially inhomogeneous, unstable and sensitive to the small changes of film deposition conditions, and LC wetting properties resulting in observed pretilt data spread for different experiments.

LC alignment appears due to the competition of two orienting effects. The first is an effect of the columnar SiO_2_ relief, which tends to align the LC director along the pore axis [27]. The second is the effect of the averaged plane SiO_2_ surface tending to homeotropically orient the LC. The result of that competition depends on the relief scale relative to the LC molecule length.

The LC pretilt is somewhat less than β_SiO2_ at 50° < α < 75° because the surface relief is weakly pronounced. At α > 75°, a well-formed columnar structure dominates LC orientation; however, β_LC_ is somewhat larger than β_SiO2_ which can be caused by good wettability of the orienting surface by the LC [5].

## Conclusion

To summarize, we investigated the anisotropic optical properties of thin SiO_2_ layers as a function of the deposition angle. The optical properties of columnar SiO_2_ were modeled by effective media theory and depolarization factors were used to describe birefringence. Birefringence increased with the deposition angle, while refractive indices decreased. It is shown that ellipsometry can obtain the orientation of the SiO_2_ columns via index ellipsoid measurements. The pretilt of LC molecules aligned by obliquely deposited SiO_2_ layers was measured and was found to be close to the SiO_2_ column tilt for deposition angles larger than 75°.
